# Editorial: Gene regulatory networks involved in the molecular response to drought, salt and osmotic stresses in crops

**DOI:** 10.3389/fpls.2023.1328377

**Published:** 2023-11-07

**Authors:** Teresa Docimo, Elide Formentin, Elena Baldoni

**Affiliations:** ^1^ National Research Council (CNR), Institute of Biosciences and Bioresources (IBBR), Portici, Italy; ^2^ Synthetic Biology and Biotechnology Unit, Department of Biology, University of Padua, Padua, Italy; ^3^ Botanical Garden, University of Padua, Padua, Italy; ^4^ National Research Council (CNR), Institute of Agricultural Biology and Biotechnology (IBBA), Milan, Italy

**Keywords:** drought, salt stress, stress tolerance, crops, climate change, stress signalling, transcription

Water shortage and high salinity are global concerns that affect crop production worldwide, with a great impact especially in semi-arid and arid regions. Extreme events due to climate changes are worsening the impact of environmental cues on crop yield. Hence, the development of tolerant genotypes is a primary goal of breeding programs. The physiological and molecular bases of abiotic stress response are not well dissected in crops and few applications of the related knowledge are available to this purpose. In this context, the articles presented in this Research Topic provide novel insights into the plant response to water and salt stresses. The presented works concern: i) the model species *Arabidopsis thaliana*, in a perspective of translational biology, ii) two stress-tolerant wild species (*Aeluropus littoralis* and *Fagopyrum leptopodum*), to discover novel tolerance genes, and iii) two primary crops for human nutrition, wheat (*Triticum aestivum* L.) and tomato (*Solanum lycopersicum* L.).

Stress responses in plants encompass a multitude of molecular mechanisms that range from the perception of stress to the reprogramming of the transcriptional machinery, leading to a variety of biochemical and physiological adaptations that enable plants to survive and thrive. The initial step in this intricate network involves the perception of stress. Second messengers, including calcium ions (Ca^2+^) and reactive oxygen species (ROS), play vital roles in translating stress perception into coordinated responses within plants. Chloroplasts have a key role in sensing stress and initiating retrograde signals to the nucleus, influencing stress-induced Ca^2+^ fluctuations by moving Ca^2+^ from the cytosol into the stroma. The involved transporters are currently unknown in crops. Recently, a member of the Ca^2+^ uniporters MCU family, named chloroplast MCU (cMCU), has been identified in Arabidopsis and characterized for its role in regulating Ca^2+^ uptake into chloroplasts during osmotic and oxidative stress. The work of Corti et al. delves into the molecular networks governed by cMCU, revealing that in *cmcu* mutant plants the ABA/IAA ratio is higher than in wild-type, photosynthesis-related proteins are induced, thus explaining the semi-closure of stomata and sustained photosynthesis previously reported. Moreover, they hint at a different regulation of the plastidial Ca^2+^ sensor CAS, suggesting a potential divergence in retrograde signalling in *cmcu* mutants.

Abiotic stresses cause ROS accumulation in plant cells. When appropriately managed by scavenging systems (*e.g.*, catalases, superoxide dismutase, and peroxidases), stress-induced ROS act as signalling molecules that modulate gene expression and enhance stress tolerance. Xiao et al. conducted a functional analysis of TaBAS1, a typical 2-Cys peroxiredoxin found in wheat chloroplasts. The authors examined the role of TaBAS1 on salt tolerance in wheat. In particular, they found that its activity is related to that of plasma membrane NADPH oxidases (NOXs), which are involved in the control of ROS levels. TaBAS1 overexpression in wheat enhances salt tolerance without compromising crop yield, suggesting the potential for selecting high-yield and salt-tolerant wheat varieties.

The role of transcription factors (TFs) in stress response is well documented, and the identification of novel TF able to confer tolerance in crops is an important topic. Wang et al. studied the TF FlbZIP12 in *Fagopyrum leptopodum*, a drought-tolerant wild relative of buckwheat, a highly demanded gluten-free cereal. *FlbZIP12* expression is induced by abiotic stressors, including abscisic acid (ABA), osmotic and salt treatments. When overexpressed in buckwheat root hairs, FlbZIP12 promoted drought tolerance through increased activities of ROS scavengers and the expression regulation of stress-related genes. In particular, the interaction of FlbZIP12 with SnRK2-like protein kinases influences the expression of both stress-responsive genes (*i.e.*, *RD29B* and *ERD1*) and flavonoid biosynthetic genes. Overall, the authors showed that FlbZIP12 is a positive regulator of ABA signalling pathway, suggesting that it is a promising candidate for improving drought tolerance in buckwheat.

Transcriptional reprogramming occurs during stress response and transcriptomic studies can significantly support the identification of genes and regulatory networks regulating plant responses to environmental fluctuations. Pirona et al. analyse the transcriptomic changes that occurs in both root and leaves of tomato plants subjected to osmotic stress. This work shed new light on the regulatory networks occurring under osmotic stress and identified hub genes, including TFs, that putatively orchestrate the osmotic stress response in tomato and may represent potential candidates for improving stress tolerance. The findings of this work well integrate the interesting review by Rosca et al., that gives a synthetic insight of the recent scientific advances related to morphological, physiological, biochemical and molecular changes in tomato plants subjected to high salinity. In particular, the authors summarize the results obtained in 98 studies that have been sifted according to Preferred Reporting Items for Systematic Reviews and Meta-Analyses (PRISMA) guideline. The authors underline how the effect of salinity depends on the kind of imposed treatment and on the different behaviour of the considered genotypes. Finally, the review proposes specific strategies for alleviating the effect of salinity on tomato, including genetic modification, CRISPR-Cas technology, QTL mapping and gene pyramiding.

Growth and development are strongly affected by abiotic stresses and the exploit of genes with a key role in these processes can be a successful strategy for maintaining high growth rate and yield in crops. Laccases are plant enzymes with essential functions in plant development, since they are involved in lignin polymerization and deposition in the plant cell wall, and consequently in the response to environmental stresses. Hashemipetroudi et al. identified and characterized fifteen laccase-encoding genes, named AlLAC, of the halophyte species *A. littoralis*. Interaction network analysis based on Arabidopsis orthologues supported the involvement of laccases in diverse cellular processes, especially those related to cell wall biogenesis. The authors also examine the expression patterns of AILAC genes in root and leaf and under stress conditions, envisaging their function in improving tolerance to adverse environmental cues.

Taken together, the studies collected in this Research Topic contribute to enhance our knowledge about plant response to environmental stresses. Researchers have transitioned from cellular-level signalling to system-wide approaches, moving from single-gene studies to ‘omics’ research. These findings provide novel elements for dissecting the mechanisms underlying stress tolerance, a critical endeavour in the face of climate change and increasing food production demand.

## Author contributions

TD: Writing – original draft, Writing – review & editing. EF: Writing – original draft, Writing – review & editing. EB: Conceptualization, Writing – original draft, Writing – review & editing.

